# Neuromarkers from Whole-Brain Functional Connectivity Reveal the Cognitive Recovery Scheme for Overt Hepatic Encephalopathy after Liver Transplantation

**DOI:** 10.1523/ENEURO.0114-21.2021

**Published:** 2021-08-18

**Authors:** Yue Cheng, Wen Shen, Junhai Xu, Rachel C. Amey, Li-Xiang Huang, Xiao-Dong Zhang, Jing-Li Li, Cameron Akhavan, Ben A. Duffy, Julia Pia Simon, Wenjuan Jiang, Mengting Liu, Hosung Kim

**Affiliations:** 1 Department of Radiology, Tianjin First Center Hospital, Tianjin 300192, People’s Republic of China; 2 Tianjin Key Laboratory of Cognitive Computing and Application, School of Artificial Intelligence, College of Intelligence and Computing, Tianjin University, Tianjin 300350, People’s Republic of China; 3 U.S. Army Research Institute for the Behavioral and Social Sciences, Fort Belvoir, Virginia 22060-5610; 4 College of Pharmacy, Western University of Health Sciences, Pomona, California 91766-1854; 5 USC Mark and Mary Stevens Neuroimaging and Informatics Institute, Keck School of Medicine of USC, University of Southern California, Los Angeles, California 90033

**Keywords:** connectome-behavior predictive mapping, functional network, liver transplantation, neuropsychological assessment, overt hepatic encephalopathy

## Abstract

Neurocognitive impairment is present in cirrhosis and may be more severe in cirrhosis with overt hepatic encephalopathy (OHE). Liver transplantation (LT) can restore liver function, but how it reverses the impaired brain function is still unclear. MRI of resting-state functional connectivity can help reveal the underlying mechanisms that lead to these cognitive deficits and cognitive recovery. In this study, 64 patients with cirrhosis (28 with OHE; 36 without OHE) and 32 healthy control subjects were recruited for resting-state fMRI. The patients were scanned before and after LT. We evaluated presurgical and postsurgical neurocognitive performance in cirrhosis patients using psychomotor tests. Network-based statistics found significant disrupted connectivity in both groups of cirrhotic patients, with OHE and without OHE, compared with control subjects. However, the presurgical connectivity disruption in patients with OHE affected a greater number of connections than those without OHE. The decrease in functional connectivity for both OHE and non-OHE patient groups was reversed after LT to the level of control subjects. An additional hyperconnected network (i.e., higher connected than control subjects) was observed in OHE patients after LT. Regarding the neural–behavior relationship, the functional network that predicted cognitive performance in healthy individuals showed no correlation in presurgical cirrhotic patients. The impaired neural–behavior relationship was re-established after LT for non-OHE patients, but not for OHE patients. OHE patients displayed abnormal hyperconnectivity and a persistently impaired neural–behavior relationship after LT. Our results suggest that patients with OHE may undergo a different trajectory of postsurgical neurofunctional recovery compared with those without, which needs further clarification in future studies.

## Significance Statement

After liver transplantation, brain functional impairment induced by cirrhosis shows some recovery in terms of both brain functional connectivity and cognitive task performance. However, cirrhotic patients with and without OHE present different recovery paths. We show that a hyperconnected network emerges for OHE patients only after liver transplantation, within which the connectivity is abnormally higher than that in healthy control subjects. Furthermore, the predictive power of the neuromarkers for cognitive task performance returns to normal in non-OHE patients only, but not in OHE patients. These findings suggest that OHE patients may exhibit functional reorganization in brain functional MRI after liver transplantation. That is, the brain connectivity responsible for cognitive task performance alters, and the brain–behavior relationship is reshaped.

## Introduction

Overt hepatic encephalopathy (OHE) is one of the most prominent complications in cirrhotic patients. It decreases a patient’s quality of life, in addition to lowering the survival rate. Neurocognitive impairment is present in cirrhosis and occurs along a continuous spectrum. These impairments are accelerated by episodes of OHE (Bajaj et al., 2010), which can often irreversibly impair attention, learning ability, and executive function (Campagna et al., 2014). Studies have demonstrated (Bajaj et al., 2010; Umapathy et al., 2014) poorer neurocognitive performance in patients with OHE than in those without.

Liver transplantation (LT) is the only effective treatment for end-stage cirrhosis (Cárdenas and Ginès, 2011). Successful LT can restore liver function completely. However, to what extent cognitive dysfunction can be recovered, and to what extent OHE can impact the recovery process, is still unclear (Sotil et al., 2009; Ahluwalia et al., 2016; Cheng et al., 2017).

Neurocognitive deficits and recovery are usually assessed by extensive psychometric neurocognitive testing for cirrhosis studies. Previous studies (Bajaj et al., 2009; Sotil et al., 2009; Campagna et al., 2014) consistently demonstrate that patients with cirrhosis, both with and without OHE, produce low performance in the Mini-Mental State Exam, the Number Connection Test (NCT), and the Digit Symbol Test (DST). This suggests that the impairment led by cirrhosis is not limited to a specific functional domain, but rather includes a wide range of cognitive functions including short-term memory (recall), attention, language, comprehension, motor skill, as well as executive functions. These impairments were reflected by brain atrophy and decreased functional connectivity (FC; Chen et al., 2012). Functional MRI, particularly resting-state functional connectivity (Qi et al., 2012, 2014; Chen et al., 2016; Cheng et al., 2017; Zhang et al., 2017a,b), could help to further unravel the underlying mechanisms that lead to these cognitive deficits and recovery. Several studies have investigated the association between impaired cognitive functions and the functional connectivity related to these functions using hypothesis-driven approaches (Qi et al., 2012; García-García et al., 2018). However, which localized functional connectivity corresponds to which cognitive functions is still not well established (Poldrack, 2010; Barch et al., 2013; Poldrack and Yarkoni, 2016). Furthermore, studies of patients with various brain injuries have shown evidence that functional hyperconnectivity or brain reorganization may occur in response to the initial brain injury, leading to broader connectivity changes beyond the changes in the relevant brain regions (Castellanos et al., 2010; Grefkes and Ward, 2014; Bharath et al., 2015; Bernier et al., 2017). This makes it difficult for investigators to arrive at a clear conclusion for their neuroimaging data when applying hypothesis-driven approaches, where only hypothetically relevant functional regions are investigated. Hence, searching for possible biomarkers across the whole brain network, via data-driven approaches, without restriction within known brain functional regions, connections, and functional networks, is critical to understanding the cirrhosis-associated neurocognitive impairments and recovery.

Associated brain biomarkers can often make inferring behavior from functional connectivity difficult (Woo et al., 2017). The biomarkers are typically found using a hypothesis driven approach, based on findings from previous related studies (Yarkoni et al., 2011). To circumvent these potential problems, further analyses need to be conducted that require a technique to understand intrinsic network properties, an approach that is capable of isolating unique patterns characterizing cognitive performance.

In this study, we used a data-driven approach that combined a cognitive task with whole-brain FC to provide a new perspective on cognitive deficits and recovery for OHE and non-OHE cirrhotic patients before and after transplant surgery. We evaluated neurocognitive deficits in cirrhotic patients and recovery after LT using a standard psychomotor cognitive task that is reliant on a variety of functional neural components like sensorimotor, attention, memory, and executive functions (Miyake et al., 2000). To avoid limiting our study to the analysis of brain regions, connections, and functional networks hypothetically related to neurocognitive impairment in patients with cirrhosis, we used “connectome predictive mapping” (CPM) to identify unique patterns characterizing cognitive performance. The CPM searches for all possible pairs of brain regions and their associated connectivity values, consequently constructing a connectome model that optimizes the association with behavioral scores (Finn et al., 2015; Rosenberg et al., 2017; Shen et al., 2017). The predictive power and the robustness of the connectome was further validated using cross-validation on novel/unseen subjects. As such, CPM is a data-driven prediction method that can identify what aspects of network properties characterize cognitive task performance in individuals. Using CPM, we address questions such as the following. (1) Compared with healthy control subjects (HCs), are the disrupted or altered functional connections in cirrhotic patients with OHE, or in both cirrhotic groups regardless of OHE? (2) Are these connections recovered, reversed, or reorganized after LT treatment? (3) Are the re-established connections after LT the same as for control subjects or are they different from control subjects because of brain reorganization? (4) Is the normal relationship that is observed between a healthy brain and cognitive performance impaired in presurgical cirrhotic patients, and is this recovered after LT?

## Materials and Methods

### Subjects

**Figure 1.**
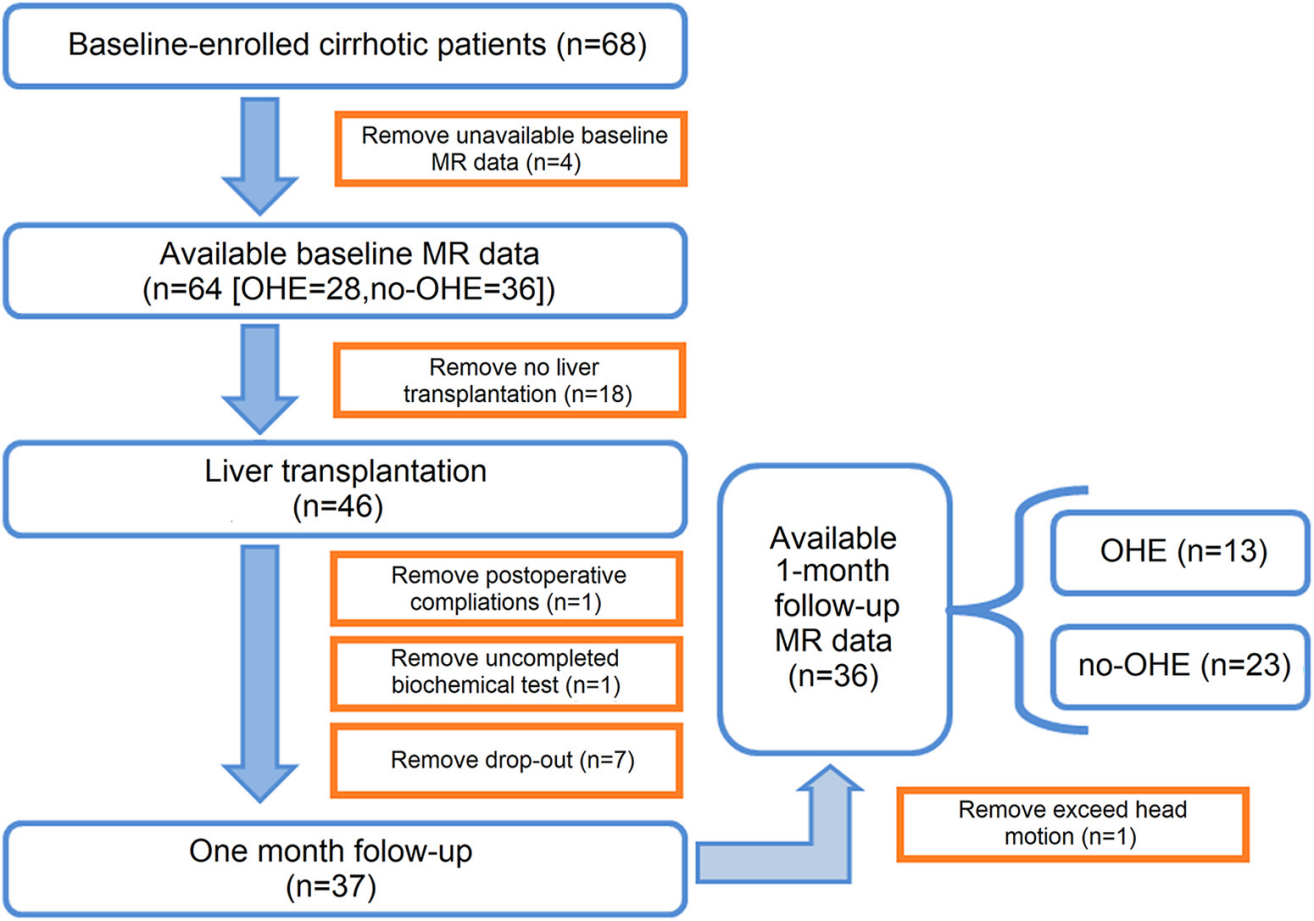
Subjects involved in this study. Sixty-eight cirrhotic patients were enrolled in this study, and a subset of patients was removed from the subject pool for the following reasons: unavailable baseline MR data, not undergoing LT, postoperative complications, incomplete biomedical examination, dropping out, or having excess head movement (see panels for exact numbers). In total, 28 patients with OHE and 36 without OHE were analyzed before LT; 13 patients with a history of OHE and 23 patients without a history of OHE participated in the study after the LT operation.

In this prospective study, approval was obtained from the Ethics Committee of institutional review board from Tianjin First Center Hospital. All subjects provided written informed consent before being included in the study. From November 2013 to January 2018, 64 patients with end-stage cirrhosis (28 patients with a history of OHE episodes; 36 patients without a history of OHE episodes) scheduled to undergo LT were recruited from the Department of Transplantation Surgery in Tianjin First Center Hospital. Thirty-two of the 64 patients have been previously reported (Cheng et al., 2015; Zhang et al., 2017b) investigating regional functional activity, whereas in this study we evaluated functional connectivity and specifically its predictive power for behavior (i.e., the psychomotor cognitive performance score). LT candidates who completed all necessary laboratory examinations, neuropsychological tests, and baseline MRI were included. Participants were excluded if they reported the following: (1) a history of drug or alcohol abuse; (2) the presence of any noticeable brain lesions on conventional MR, such as a tumor or stroke; (3) any major neurologic or psychiatric disorders; (4) history of liver cancer; (5) previous liver or other organ transplantation; and (6) head motion of 1.5 mm or 1.5° during MRI. The etiology of the cirrhotic patients included type B hepatitis (*n* = 35), type C hepatitis (*n* = 15), primary biliary cirrhosis (*n* = 8), and cryptogenic cirrhosis (*n* = 6). Thirty-six of these patients eventually received successful LT and completed the 1-month follow-up examination. Included patients had no complications such as acute transplant rejection, liver failure, severe biliary complications, or any neurologic complications, such as alterations in mental status, seizures, and focal motor deficits. A detailed flowchart of this study is provided in Figure 1.

Thirty-two age- and sex-matched HCs were recruited from the local community. All HCs had no diseases or history of liver or neurologic diseases. All HCs were self-identified as right handed, with normal sight, and had completed neuropsychological tests (see details in Neuropsychological tests).

### Laboratory examinations

All patients completed blood laboratory tests to evaluate liver function 1 week before their MRI scan, both before and 1 month after LT. These tests included prothrombin time, albumin, and total bilirubin. For the preoperative patients, their Child–Pugh score was used to assess liver dysfunction. Venous blood ammonia was also tested for cirrhotic patients. No blood laboratory tests were performed for HCs.

### Neuropsychological tests

All patients and HCs underwent two typical neuropsychological tests to evaluate cognitive function before the MRI scan. These tests included the NCT type A (NCT-A; abbreviated as NCT in all text below) and the DST (Weissenborn et al., 2001). The NCT and DST are part of the Psychometric Hepatic Encephalopathy Score (Ferenci et al., 2002; Weissenborn et al., 2005) and are ideal for this study as they are supported by a variety of functional neural components given their reliance on different sensorimotor, attention, memory, and executive functions (Miyake et al., 2000). The DST and NCT have undergone repeated and rigorous psychometric validation such as test–retest reliability and discriminant validity in a range of patient samples (Matarazzo and Herman, 1984; Jaeger, 2018; Zeng et al., 2020). In the NCT task, participants were required to connect randomly placed figures in order as quickly as possible to measure psychomotor speed and attention (Bajaj et al., 2009). Completion time is indicative of performance, and worse performance is indicated by a longer completion time. The DST is a measure of complex visuomotor tracking and learning, which emphasizes sustained attention, response speed, and visuomotor integration (Bajaj et al., 2011). Digits from one to nine and corresponding symbols are displayed in front of the subjects, who are then asked to fill in the blanks with the symbol that matched each digit. Participants have 90 s to complete this task. The number of correctly transcribed symbols is used as an indicator of performance—lower scores indicate underperformance.

### MRI data acquisition

The images were collected at Tianjin First Central Hospital using a 3.0 T magnetic resonance scanner (TIM-Trio, Siemens Medical Solutions) and an eight-channel head coil. For functional images, blood oxygen level-dependent single-shot echoplanar sequence parallel to the anterior commissure–posterior commissure plane [repetition time, 2500 ms; echo time, 30 ms; flip angle, 90°; field of view, 220 × 220 mm^2^; matrix, 96 × 96; iPAT (integrated parallel imaging technique) factor, 2; number of slices, 40; slice thickness, 3 mm; intersection gap, 0.3 mm; 200 volumes; acquisition time, 8.5 min] was performed. During the scan, all subjects were instructed to close their eyes and remain awake, they were asked to think of nothing in particular. For structural images, a sagittal 3D T1-weighted MPRAGE (magnetization-prepared rapid acquisition gradient echo) sequence was used with the following settings: repetition time, 1900 ms; echo time, 3 ms; inversion time, 900 ms; flip angle, 9°; number of slices, 176; slice thickness, 1 mm; matrix, 256 × 256. During the whole scanning period, foam pads were used to reduce head movement and ear plugs were used to reduce scanner noise. All acquired images were visually inspected and confirmed by an independent radiologist to be free of any problems occurring during image acquisition and of any significant structural lesions.

### Data preprocessing

Functional imaging data were preprocessed using SPM12 software (https://www.fil.ion.ucl.ac.uk/spm/). For each participant, the first 10 volumes were removed to allow for dynamic equilibrium and adaption to the scanning circumstances. The remaining functional images were corrected for time delays between slices using slice timing. A six-parameter rigid body transformation in the realignment analysis was performed to correct head motion. Participants with a translation exceeding 3 mm and a rotation of >1.5° were excluded for further analyses. In the present study, no such participants were removed. Each individual subject’s structural image was then segmented into gray matter, white matter (WM), and CSF for normalization after coregistration to the mean functional image. All functional images were spatially normalized to the standard MNI (Montreal Neurologic Institute) space using the generated parameters at an isotropic voxel size of 3 mm. Finally, normalized functional images were smoothed with a 4 mm full-width at half-maximum Gaussian filter aiming to improve the signal-to-noise ratio. A linear detrended and temporally bandpass filtered (0.01 Hz < *f* < 0.08 Hz) procedure was performed to reduce the effects of low- frequency drift and high-frequency physiological noises (Biswal et al., 1995). Several sources of spurious variance along with their temporal derivative were used to reduce the physiological noise and remove artifacts by the following linear regression: averaged signals from WM, CSF, and six head motion parameters.

### Whole-brain functional connectivity analysis

To obtain the whole-brain FC matrix, a prior Anatomical Automatic Labeling template was used to divide the whole brain into 116 anatomic regions of interest (ROIs), including 78 cortical, 12 subcortical, and 26 cerebellar regions (Tzourio-Mazoyer et al., 2002). A representative time series was extracted by averaging the time series of all voxels within each ROI. Then, Pearson’s correlation analyses were performed between each pair of ROIs to calculate the correlation coefficients, followed by the normalization with a Fisher *z* score transformation. A symmetric functional connectivity matrix (116 × 116) was generated for each subject. The triangular portion of the adjacency matrix was extracted and transformed to a vectoral feature space with 6670 dimensions.

### Network-based statistic

To localize specific pairs of brain regions between which functional connectivity was altered in cirrhotic patients, we used the network-based statistic (NBS) approach (Zalesky et al., 2010). We first performed two-sample one-tailed *t* tests in an element-by-element manner on those connections that were significantly nonzero (*p* < 0.05, Bonferroni corrected) in at least one participant. Then, a primary threshold (*p* < $1\times 10^{-4}$ in this study; Wang et al., 2013) was applied to define a set of suprathreshold links within which any connected components and their size (defined as the number of links included in these components) were determined. To estimate the significance for each component, a null distribution of connected component size was derived empirically using a nonparametric permutation approach (10,000 permutations). For each permutation, all subjects were reallocated randomly into two groups, and two-sample one-tailed *t* tests were conducted for the same set of connections mentioned above. The same primary threshold (*p* < $1\times 10^{-4}$) was then used to generate suprathreshold links within which the maximal connected component size was recorded. Finally, for a connected component of size M (i.e. number of edges) found in the right grouping of control subjects and patients, the corrected *p* value was determined by calculating the proportion of the 10,000 permutations for which the maximal connected component was larger than M.

NBS was first applied to compare the FC differences between control subjects and pre-LT patients to show the disrupted connectivity induced by cirrhosis. NBS was then applied to compare control subjects and post-LT patients to show whether there was still impaired FC after the LT. Finally, NBS was conducted to compare pre-LT and post-LT patients to investigate the FC recovery and hyperconnectivity.

### Connectome behavior predictive mapping

Connectome behavior predictive mapping and all statistics were assessed using MATLAB. To investigate reliable biomarkers in predicting task performance in resting-state whole-brain FC, a completely data-driven approach of CPM was used for HCs. CPM searched all possible pairs of regions and their associated connectivity values, constructing a model (the “connectome”) that maximally fitted behavioral scores (Finn et al., 2015; Rosenberg et al., 2017; Shen et al., 2017) using cross-validation. Specifically, linear regressions were run between each edge in the connectivity matrix in the resting state and DST/NCT scores. The resulting *p* value for each regression was recorded in a 116 × 116 symmetric matrix, amounting to 6670 different linear regression significance values. To find the most meaningful associations between specific connectivity and the task performance, the resulting *r* values were held to a statistical threshold of *p* < 0.01 and separated into positive tails (edges whose connectivity strength indexed higher performance scores across subjects) and negative tails (edges whose connectivity strength indexed lower performance scores across subjects). Positive and negative values in CPM were defined as a Pearson’s correlation coefficient *r* value in terms of their correlation with behaviors, regardless of better or worse performance. In our psychomotor cognitive tasks, a higher DST score represented better performance, whereas a higher NCT score represented worse performance. Therefore, a positive *r* value in CPM for the DST score indicates stronger functional connectivity related to better cognitive performance. Whereas a negative *r* value for the NCT score indicates stronger functional connectivity related to better performance.

A single summary statistic, network strength, was used to characterize each participant’s degree of connectivity in the positive and negative tails. Positive network strength was calculated by averaging the edge strengths (Fisher-normalized *r* values) from a participant’s connectivity matrix in the edges of the positive tail, and negative network strength was calculated by averaging the *r* values of the edges in the negative tail. Finally, network strength was used to predict performance scores across subjects using fivefold cross-validation (Rosenberg et al., 2016; Liu et al., 2021). Specifically, all control participants were randomly split into five subgroups. Among the five groups, one group was selected as the test dataset and the remaining four groups were used to train a CPM model to search for predictive functional connectivity for each of the cognitive task scores (DST, NCT). For the training, a linear regression model was used to fit the average value of obtained functional connectivity with each cognitive performance score. This trained model was then applied to predict performance scores in the test dataset. This process repeated 5 times until all 5 groups were tested. To validate the predictive accuracy and the robustness of the functional connectivity identified by CPM models, we correlated predicted performance scores with the observed performance scores for all participants. If the correlation was reported to be significant, then the consensus of functional connectivity maps obtained from all of the five CPM models was collected as the connectome predictor.

Meaningful connectivity-based neuromarkers discovered by CPM in control subjects were then applied in cirrhotic patients before and after LT as a *post hoc* analysis to evaluate whether the network can still predict subjects’ task performance, which subsequently evaluates the recovery of brain–behavior relations.

### Data availability

Anonymized data will be shared by request from any qualified investigator who provides a methodologically sound proposal, or for the purpose of replicating procedures and results presented in the present study. The code/software described in the article is freely available online at https://github.com/bigting84/Cirhosis.

## Results

### Demographics and clinical data

Demographics and clinical data for all subjects are summarized in Table 1. There were no meaningful differences in sex, age, or education level among OHE, non-OHE, and HC groups (two-way *t* test, *p* values > 0.05). For both the non-OHE and OHE groups, liver function improved significantly (albumin, total bilirubin, blood ammonia, *p* ≤ 0.01) or showed a tendency toward restoration (prothrombin time) 1 month after LT (Table 1).

**Table 1:** Demographic, neuropsychological, and biochemical information from the dataset

Protocols	HCs (*n* = 32)	Pre-LT (*n* = 64)		Post-LT (*n* = 36)		*p* Value
		non-OHE (*n* = 36)	OHE (*n* = 28)	non-OHE (*n* = 23)	OHE (*n* = 13)	
Sex						0.841^*a*^
Male	21	24	20	16	10	
Female	11	12	8	7	3	
Age (years)	51.2 ± 7.2	48.1 ± 9.9	49.1 ± 8.6	47.4 ± 9.4	49.1 ± 9.3	0.272^*b*^
Education (years)	12.7 ± 2.6	11.7 ± 2.8	12.4 ± 3.0	12.2 ± 2.5	12.6 ± 3.9	0.436^*b*^
NCT (s)	40.4 ± 10.2	56.5 ± 32.1	59.8 ± 22.8	44.5 ± 21.8	45.0 ± 15.6	0.009^*c*^/0.007^*d*^ 0.357^*e*^/0.253^*f*^
DST (score)	47.8 ± 10.5	39.2 ± 13.7	34.9 ± 16.5	48.3 ± 15.0	42.3 ± 17.4	0.010^*c*^/0.006^*d*^ 0.897^*e*^/0.200^*f*^
Prothrombin time (s)		17.8 ± 6.3	17.4 ± 4.5	14.8 ± 7.7	13.3 ± 6.4	0.105^*g*^/0.022^*h*^
Albumin (mg/dl)		33.2 ± 8.3	31.9 ± 5.8	40.5 ± 6.0	39.3 ± 5.5	0.000^*g*^/0.001^*h*^
Total bilirubin (mg/dl)		78.2 ± 104.0	64.9 ± 54.5	16.3 ± 10.5	15.1 ± 8.1	0.001^*g*^/0.000^*h*^
Blood ammonia (μmol/l)		61.8 ± 21.9	84.60 ± 29.5	37.0 ± 10.0	48.2 ± 22.4	0.000^*g*^/0.000^*h*^
Child–Pugh score						
Class A		5	0	4	0	
Class B		14	6	9	2	
Class C		17	22	10	11	

Data are presented as the mean ± SD.

a The *p* value was obtained by the Pearson χ^2^ test (two-tailed).

b The *p* value was obtained by the one-way ANOVA test among the three groups (two-tailed);.

c The *p* value was obtained by the two-sample *t* test between pre-LT in non-OHE group and HCs (two-tailed).

d The *p* value was obtained by the two-sample *t* test between pre-LT in OHE group and HCs (two-tailed).

e The *p* value was obtained by the two-sample *t* test between post-LT in non-OHE group and HCs (two-tailed).

f The *p* value was obtained by the two-sample *t* test between post-LT in OHE group and HCs (two-tailed).

*^g^*The *p* value was obtained by the two-sample *t* test between post-LT and pre-LT in non-OHE group (two-tailed).

h The *p* value was obtained by the two-sample *t* test between post-LT and pre-LT in OHE group (two-tailed).

### Behavior score comparison

As expected, OHE and non-OHE groups before LT underperformed compared with HCs—patients took longer to complete NCT tasks (*p* values < 0.009) and had lower scores in DST tasks (*p* values  < 0.01). One month after LT, compared with HCs, both the OHE and non-OHE patients showed comparable performance in both DST (*p* values > 0.25) and NCT (*p* values > 0.20) tasks. The increased performance in DST scores and decreased performance in NCT scores suggests an enhancement of cognitive capability. Also, they may indicate cognitive recovery after LT for cirrhotic patients regardless of a history of OHE (Table 1).

### Disrupted functional connectivity in cirrhosis

**Figure 2.**
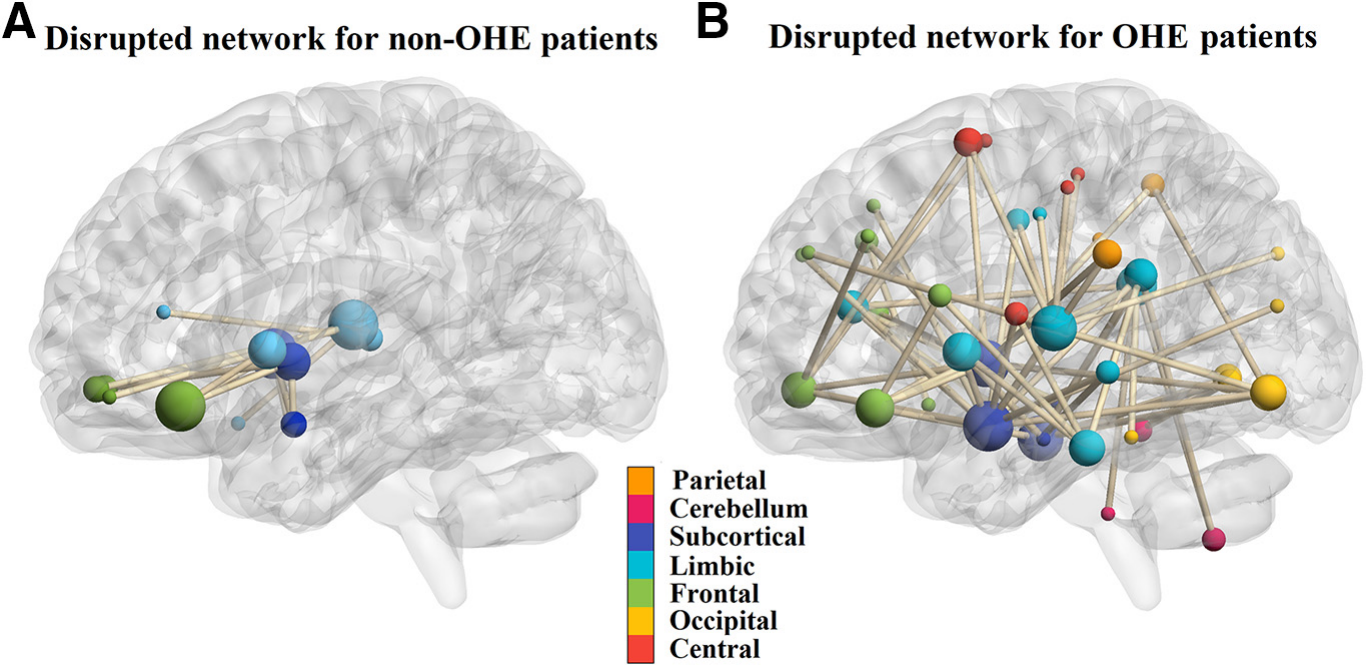
The disrupted resting-state functional networks for pre-LT non-OHE and OHE patients detected by NBS. ***A***, The region pairs showing significantly decreased functional connections in non-OHE patients compared with control subjects. These connections formed a single connected network with 16 nodes and 17 connections, which was significantly (NBS, *p* = 0.037, corrected) abnormal in the patients. The decreased connectivity was mostly found in the connections among subcortical nuclei such as amygdala, putamen, pallidum, and orbital frontal cortex. ***B***, The region pairs showing decreased functional connections in OHE patients. These connections formed a single connected network with 47 nodes and 65 connections, which was significantly (NBS, *p* = 0.006, corrected) abnormal in the patients. The connectivity was mostly found in the connections involving the following structures: subcortical nuclei (amygdala, putamen, pallidum), limbic system (temporal cortex, cingulate cortex, parahippocampal gyrus), orbital frontal cortex, and other frontal, parietal cortex and cerebellum brain regions. Functional connectivity was visualized using the BrainNetViewer toolbox in MATLAB (Xia et al., 2013), and an MNI-ICBM152 brain template (http://www.bic.mni.mcgill.ca/ServicesAtlases/ICBM152NLin2009) was used to show the brain coordinates.

NBS was first applied to compare the FC differences between control subjects and pre-LT cirrhosis. For patients without OHE, using the cluster-defining threshold of *p* < 1 × 10^−4^ (explained in Materials and Methods), a single network of 17 connections among 16 brain regions was revealed, showing decreased functional connectivity in the non-OHE group (*p* = 0.037, corrected). We found that the decreased connectivity was mostly found in the connections among subcortical nuclei such as amygdala, putamen, and pallidum as well as cortical regions including orbital fontal, insular, and cingulate cortices (Fig. 2*A*).

For OHE patients, a single network of 65 connections linking 47 brain regions was revealed with decreased functional connectivity (*p* = 0.006, corrected). The connectivity was mostly found in the connections involving the following structures: subcortical nuclei (amygdala, putamen, pallidum), limbic system (temporal cortex, cingulate cortex, parahippocampal gyrus), orbital frontal cortex, and other frontal, parietal cortex and cerebellum brain regions (Fig. 2*B*).

NBS was then applied to compare the FC differences between control subjects and post-LT cirrhosis. There were no significantly different connections found in both OHE and non-OHE patients.

### Reversed functional connectivity after LT

**Figure 3.**
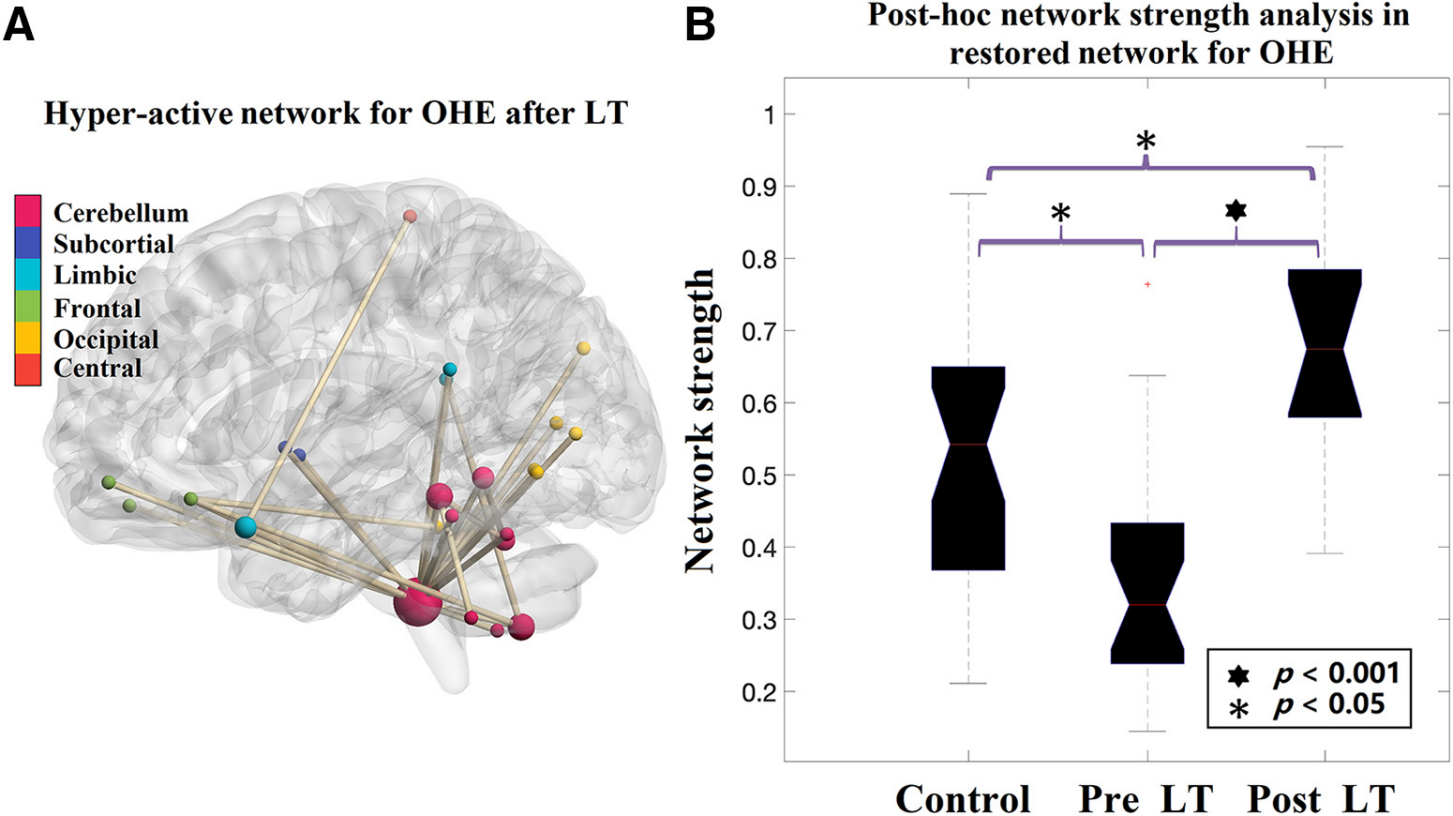
The restored resting-state functional network for post-LT OHE patients detected by NBS. ***A***, The region pairs showing increased functional connections in OHE patients after LT compared with pre-LT scans. These connections formed a single connected network with 19 nodes and 17 connections, which was significantly (*p* = 0.009, corrected) higher after LT. The increased connectivity was mostly found in the connections between cerebellum subregions, and between cerebellum and occipital cortex, orbital-frontal cortex, and limbic systems. ***B***, *Post hoc* analysis comparing the strength of the network detected in post-LT OHE patients in three groups. The strength of the network after LT was significantly higher than that in control subjects (two-sample *t* test, *p* = 0.008, corrected), suggesting an LT-induced hyperconnectivity within the detected network.

Furthermore, using the cluster defining threshold of *p* < 1 × 10^−4^, one additional network of 19 connections between 17 brain regions was revealed, showing increased functional connectivity in the OHE group (*p* = 0.009, corrected) after LT relative to the connectivity found before LT. We found that this increased connectivity was not involved in the disrupted network seen before LT, but mostly found in the connections between cerebellum subregions, and between cerebellum and occipital cortex, orbital-frontal cortex, and limbic systems (Fig. 3*A*). A *post hoc* analysis using two-sample *t* test revealed that the network strength of this network in OHE post-LT scans was significantly higher than healthy control subjects (*p* = 0.008; Fig. 3*B*), suggesting an LT-induced hyperconnectivity for OHE patients only.

### Brain–behavior model establishment in healthy control subjects: cross-group validation

A fivefold cross-validation was applied to evaluate whether the networks selected from CPM could be generalized to predict unseen subjects. This step was critical because the CPM used was constructed using healthy individuals. Once established, the selected functional connectivity would be used as a biomarker to evaluate the relationships between functional connectivity and behavioral outcomes before and after LT for the cirrhotic patient group. To this end, the positive and negative network models described in the Materials and Methods section were used.

**Figure 4.**
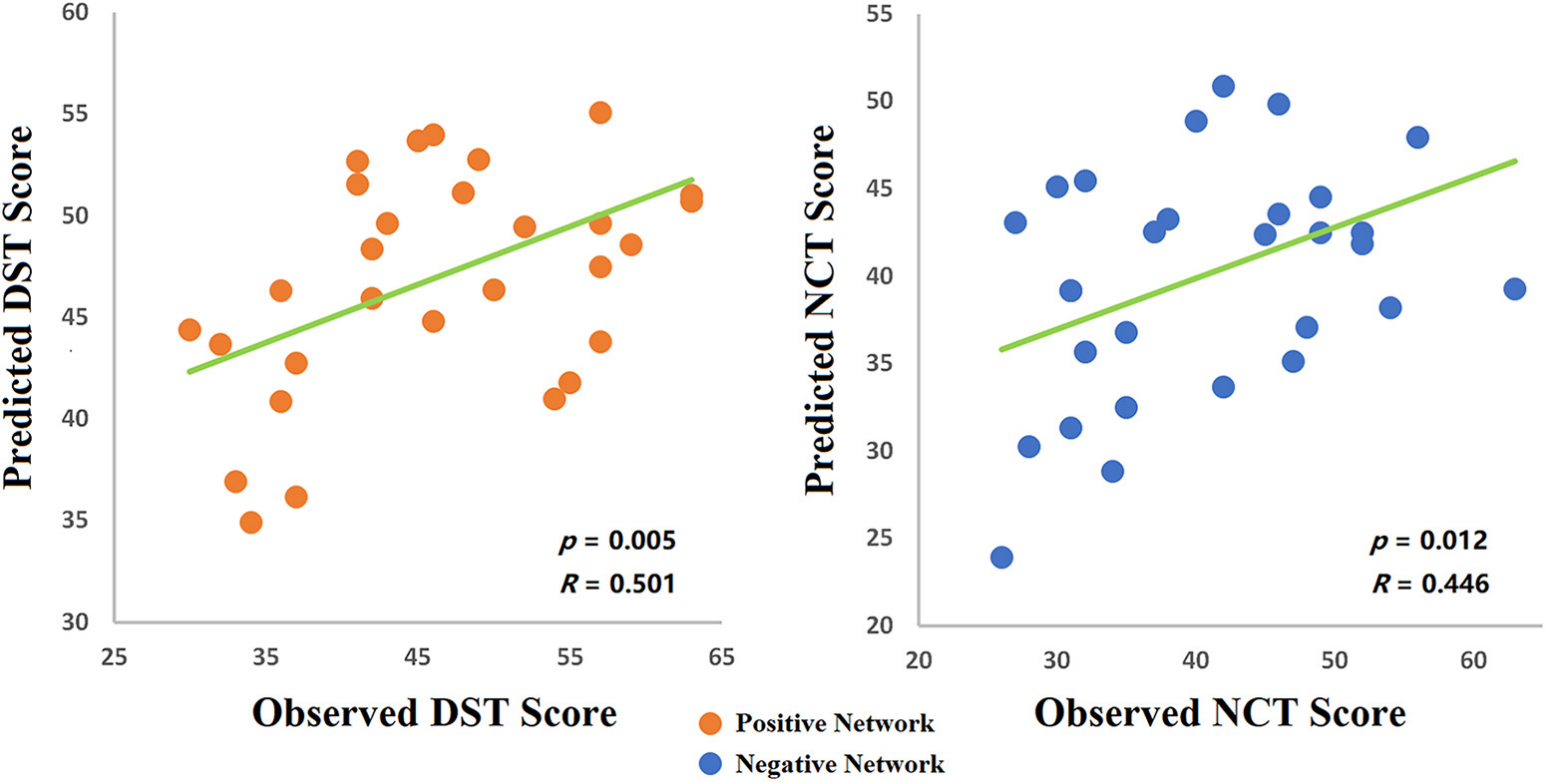
Cross-validation for the CPM. Functional connectivity models predicting cognitive task performance in healthy individuals. Scatter plots show correlations between observed performance scores and predictions by positive (left, for DST) and negative (right, for NCT) networks. Network models were iteratively trained on resting-state data from training subjects in the control group and tested on resting-state data from the individuals that were left out. For negative networks in the DST task and positive networks in the NCT network, no meaningful correlations were found.

In healthy control subjects, our results showed that the CPM models trained on positive networks predicted the DST task scores of unseen individuals (correlation between predicted and observed DST scores: *r* = 0.501, *p* = 0.005), and the models trained on negative networks predicted NCT scores (correlation between predicted and observed: *r* = 0.446, *p* = 0.012; Fig. 4). Therefore, the positive network model predicting DST scores and the negative network model predicting NCT scores were further used to explore brain–behavior relationships in OHE and non-OHE patients.

**Figure 5.**
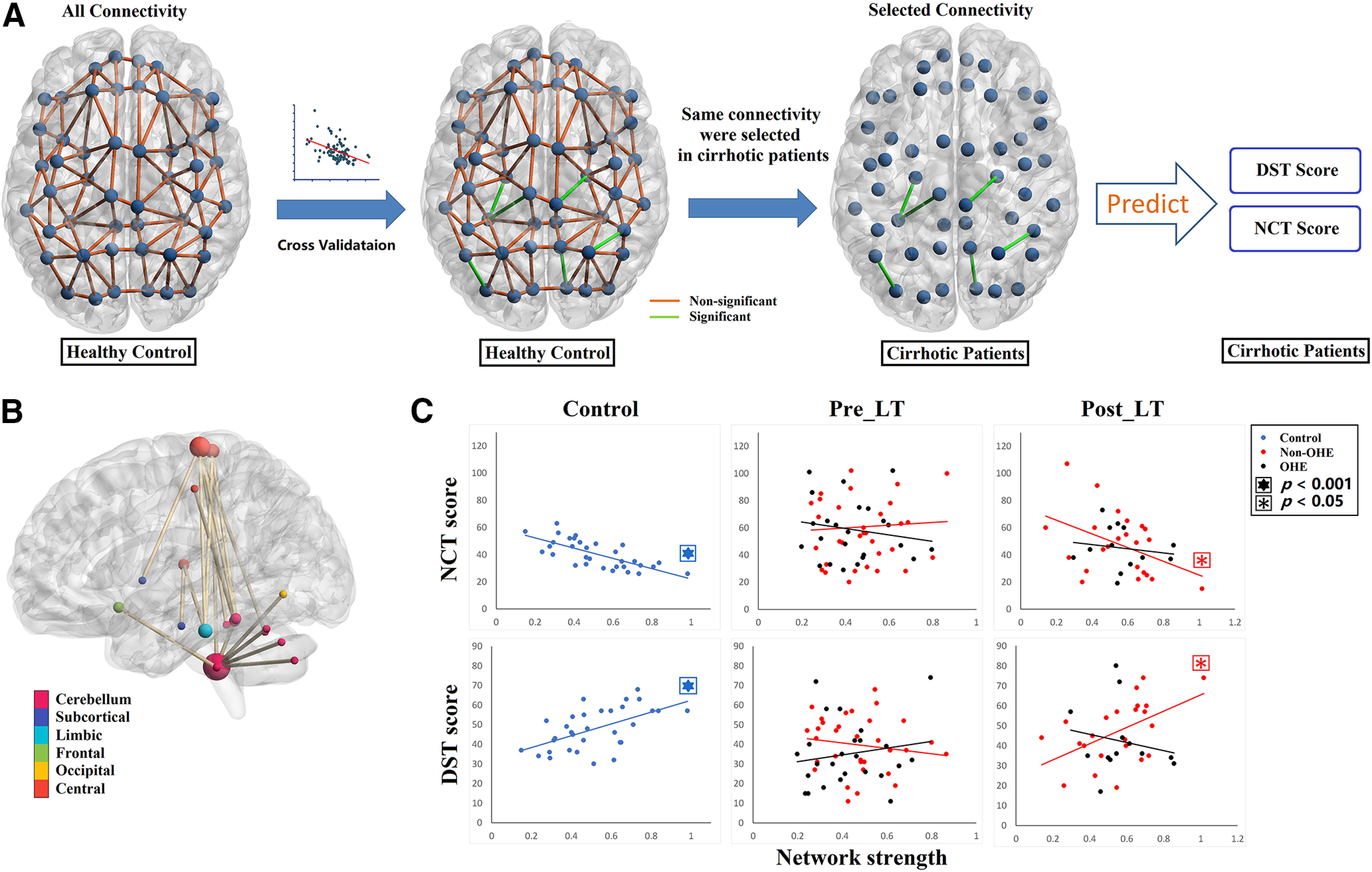
The connectivity-based neuromarkers obtained using connectome–behavior predictive mapping. ***A***, Connectome–behavior predictive mapping found every single connection that had a meaningful relationship (*p* < 0.01) to NCT/DST scores in HCs using cross-validation. This meaningful connectivity was then used to predict NCT/DST performance for patients before and after LT. ***B***, The region pairs with functional connections negatively predicting NCT scores and positively predicting DST scores in control subjects. These connections formed a single connected network with 19 nodes and 18 connections, and we call this network the PMN. The predictive connectivity in PMN was mostly found in the connections linked to cerebellum subregions and paracentral cortex. ***C***, *Post hoc* analysis comparing the predictive power of PMN network strength for NCT and DST scores in both pre-LT and post-LT scans. Before LT, network strength in PMN did not predict NCT/DST scores for both OHE and non-OHE patients. After LT, however, the cognitive test scores were predicted by PMN network strength when analyzing non-OHE patients only (DST: *r* = 0.517, *p* = 0.011; NCT: *r* = −0.424, *p* = 0.049), but not OHE patients.

Because the connections selected in each training cycle (among the five folds) were not completely identical, we extracted the connections that consistently predicted cognitive test performance in all cycles. In this manner, 6 connections from the positive network predicting DST and 12 connections from negative network predicting NCT were selected (Fig. 5*B*). The DST and NCT represented similar cognitive functions, and we found that their values significantly correlated with one another (*r* = −0.781, *p* < 0.0001). Thus, we constructed a network that combined connections that were included in both NCT negative networks and DST positive networks, and we called this network the “psychomotor network” (PMN). The PMN was then used to predict cognitive performance in patients before and after LT.

### Brain–behavior relationships in patients before and after LT

For each patient, we first computed network strength by averaging the edge strengths within the PMN, and then conducted linear models relating the PMN network strength to NCT and DST scores. The PMN strength did not predict cognitive performance for pre-LT scans of either OHE or non-OHE patients (*p* values > 0.319). However, after LT, the cognitive performance scores in non-OHE patients were predicted by PMN strength when analyzing their post-LT scans (DST: *F*_(1,21)_ = 7.67, *p* = 0.011, *r* = 0.517, β = 12.58; NCT: *F*_(1,21)_ = 4.174, *p* = 0.049, *r* = −0.424, β = −21.33; Fig. 5), but the scores in OHE patients were not (*p* values  > 0.354).

### OHE impacts the neural–behavior relationship after LT

Previously, we found that the PMN strength significantly predicted cognitive performance only in healthy control subjects and non-OHE patients after LT. Accordingly, we further assessed whether OHE played a moderative role in the predictive relationship between resting-state connectivity and cognitive performance in post-LT scans. Moderated regression analyses assessed the association of PMN strength with DST and NCT scores, including OHE as a moderator. These analyses used unstandardized regression coefficients and 95% bias-corrected confidence intervals (CIs) from 10,000 bootstrap estimates (Hayes, 2017, model 3). Results revealed a significant interaction between OHE and non-OHE groups regarding the relationship between resting-state network strength and behavioral performance (*p* = 0.029; 95% CI, 1.9423, 23.2147). No such relationships between OHE and non-OHE groups were found before LT.

Similarly, moderated mediation analyses also assessed the association of network strength with NCT scores, including OHE as a moderator. Results revealed a trend of the interaction effect between OHE and non-OHE groups (*p* = 0.061) in post-LT scans. No effect was found in pre-LT scans.

## Discussion

In this study, we investigated the impairment of functional connectivity in cirrhotic patients and the recovery of impaired functional connectivity after LT by strategically using whole-brain functional connectivity comparisons and connectome–behavior predictive-mapping approaches. We found that the transplant surgery facilitated a recovery of the disrupted functional connectivity in cirrhotic patients. Furthermore, it was shown that OHE patients became abnormally hyperactive in a specific functional network after LT, which was not part of the initially disrupted network, suggesting a reorganization of the brain network in OHE patients in response to the surgery. Exploring functional connectivity in relation to psychomotor behavioral performance, we observed that the predictive relationship between functional connectivity and behavioral performance seen in control subjects was impaired in cirrhotic patients. This impaired relationship was recovered after LT in patients without OHE history only. The moderator role of OHE suggested that the presence of OHE may hinder normal brain connectivity recovery after LT in cirrhotic patients.

### Neural and cognitive recovery difference between OHE and non-OHE patients after LT

Several interesting findings were obtained from the NBS-based comparison and *post hoc* analysis among groups, as follows: (1) OHE patients exhibited a larger number of, and more widely distributed, disrupted FC than non-OHE patients, suggesting more serious brain impairment by OHE; (2) no significantly decreased FC was found in post-LT scans compared with control subjects (i.e., disrupted networks for OHE and non-OHE both recovered); and (3) hyperconnectivity was found in post-LT scans for OHE patients only, indicating the most significantly increased connections after LT were actually abnormal (higher than those in control subjects). Note that neither OHE nor non-OHE patients exhibited significantly disrupted connectivity after LT compared with control subjects. However, the NBS revealed the significantly increased connectivity in OHE patients only after LT, compared with before LT. It is not clear whether the recovery of the initially disrupted functional connectivity occurs partially or fully (for both OHE and non-OHE patients) because NBS is a relatively rigorous statistical approach and may not capture the connectivity difference between preoperative and postoperative scans. The enhanced functional connectivity identified in OHE patients was unlikely to be naturally recovered connectivity, as it was not part of the network that was initially disrupted in the patients before LT. Rather, it could be an abnormally established or reorganized network in response to LT under severe brain impairment because of the presence of OHE.

Since hyperconnectivity in response to brain injury or insult has become common in clinical brain network studies (Hillary et al., 2015; Jones et al., 2016; Kim et al., 2020), it might be inappropriate to simply assume that the hyperconnectivity after LT as a means of better recovery for OHE. Instead, hyperconnectivity might represent a reorganization of the functional network in response to the initial damage (probably irreversible) of OHE, or another source of brain insults, as an adverse surgical effect (Filippini et al., 2009; Hillary and Grafman, 2017). In other words, OHE might induce adaptive and deformed neuroplastic changes or functional reorganization during the brain recovery process following LT. However, it remains necessary to investigate the persistence of the observed hyperconnectivity and its long-term consequences, given the chronic engagement of additional neural resources (Hillary and Grafman, 2017).

Psychomotor neurocognitive tests revealed impaired behavioral performance for cirrhotic patients compared with healthy individuals, and performance improvement after LT. Additionally, our investigation of brain–behavior predictions for cirrhotic patients sheds light on the mechanism of cognitive recovery using a generalized PMN model. For example, the PMN was not a good predictor of performance before the LT surgery for cirrhotic patients regardless of OHE. After LT, the PMN became significantly associated with cognitive performance for patients without OHE, but not for those with OHE. Given that the OHE patients also showed better behavioral performance after LT while the association between PMN and cognitive performance was impaired, it is possible that their post-LT behavioral performance was driven by the newly established hyperconnectivity or the network reorganization induced by the possibly severe and irreversible damage of OHE. Furthermore, we found that one of the key regions in PMN—cerebellum—was largely involved in the hyperconnected network for OHE after LT, suggesting that the hypercerebellar network established in OHE patients after LT may dissociate the relationship between the PMN and behavioral performance that was observed in healthy individuals. However, hyperconnectivity after LT was not observed for non-OHE patients, possibly because the brain damage because of non-OHE cirrhosis was milder. Accompanied by the recovery of functional connectivity after LT, the functional role of PMN in relation to NCT and DST tasks could therefore be recovered, which in turn drove the behavioral performance for non-OHE patients.

### Functional decoding of PMN

Psychomotor ability refers to a wide range of actions involving physical movement related to conscious cognitive processing (Ackerman and Cianciolo, 2000; Kahol et al., 2008). It measures the coordination of multiple cognitive abilities (e.g., attention, visual, executive function, with motor movement). The main hubs (degree ≥ 5 within PMN) found in the PMN include paracentral cortex and posterior cerebellum (cerebellum lobe X). Paracentral cortex is located on the medial surface of the cerebral hemisphere and is the medial continuation of the precentral and postcentral gyri. Its main role is motor and sensory functions related to the contralateral lower limb. The cerebellum is mainly involved in the control and coordination of motor movement through multiple mechanisms (e.g., timing, spatial evaluation, and sensory acquisition; Stoodley and Schmahmann, 2010). Thus, paracentral cortex and cerebellum in PMN are indicative of motor regulation, which is required in psychomotor tasks. In addition, we also found the following two hubs with relative high degree (degree ≥ 2 within PMN): inferior temporal cortex and the orbitofrontal cortex. The inferior temporal cortex has been reported as an important region associated with visual functions (Woloszyn and Sheinberg, 2012), in particular, playing an important role in visual–motor coordination. The activity in the orbitofrontal cortex is associated with some higher-order cognitive functions like emotion and emotion regulation (Rolls et al., 2020), executive function (Nejati et al., 2018), reward learning (Izquierdo, 2017), and decision-making (Du et al., 2020). Particularly, a recent study also indicated that the OFC encodes decision variables and instructs sensory areas to guide adaptive behavior (Banerjee et al., 2020). In the PMN, it is possible that the OFC plays an important role in guiding adaptive behavior to achieve a cognitive task goal in an indirect and sophisticated way. Notably, recent anatomic and functional studies also demonstrated that the cerebellum is involved in a broad range of cognitive functions in addition to its historically well known association with sensorimotor control (Strick et al., 2009; King et al., 2019). For example, the cerebellum is involved in executive function (Koziol et al., 2012), attention (Osaka et al., 2004), and emotion process (Guell et al., 2018a). Particularly, the cerebellum lobe X, which is one of the main hubs in the PMN, has been suggested to be a nonmotor functional area in several recent studies (Guell et al., 2018b; Guell and Schmahmann, 2020). Rather, it is considered to be associated with visual working memory and visual recognition (King et al., 2019). The exact role of the cerebellum in the recovery of OHE still needs to be further addressed in future studies.

### Data-driven approach may find novel and robust neuromarkers

The data-driven approach based on NBS and CPM used in this study identified novel connections that were used for group comparison and predicting cognitive test outcomes. Previously, hypothesis-driven approaches (Qi et al., 2012; García-García et al., 2018) have limited the selection of the region of interest to the brain regions that are known to be associated with the given cognitive task. Data-driven feature selection methods (Zalesky et al., 2010; Rosenberg et al., 2016) like the approach adopted in the current study, do not have such a limitation. In this regard, our approach found that the neural mechanisms for the processing of cognitive tasks in healthy subjects become impaired in cirrhotic patients and can be recovered after LT in the absence of OHE. This finding implies that specific, but novel brain connections might be involved in cognitive tasks of interest for patients with cirrhosis. This is more frequently encountered in cognitive tasks that are more taxing (e.g., tasks that demand multiple cognitive resources like attention, working memory, motor, and visual-spatial components, like psychomotor neurocognitive tasks).

### Limitations and future directions

There are several limitations in the current study. The cerebellum plays an important role in fMRI studies for OHE and cirrhosis. Although the recent literature has reported that the cerebellum facilitates successful psychomotor task performance (Medina et al., 2010; Shiroma et al., 2016), the complete role of the cerebellum in the recovery of OHE still needs to be addressed further. The current investigation of the brain functional recovery process is conducted based on patients 1 month after LT. To answer whether the recovery of cognitive function, the recovery of connectivity, the observed hyperconnectivity or functional reorganization in cognitive recovery is temporary or persistent, an extended study that includes longitudinal data with a longer follow-up after LT is needed. Our data-driven method also has its own problems: it is difficult to clarify the role of the identified functional network or brain regions when their original function is not associated with the target cognitive function. Because of the modest sample size, it is possible that some of the reported effect sizes are inflated, particularly given the high-dimensional feature space; and some of the reported results may have a higher false-positive rate (Button et al., 2013). Future studies should focus on replicating the current findings with a larger sample size.

## Synthesis

Reviewing Editor: Macià Buades-Rotger, University of Luebeck Decisions are customarily a result of the Reviewing Editor and the peer reviewers coming together and discussing their recommendations until a consensus is reached. When revisions are invited, a fact-based synthesis statement explaining their decision and outlining what is needed to prepare a revision will be listed below. The following reviewer(s) agreed to reveal their identity: NONE.

Dear authors,

Thanks for your interesting work on neural predictors of cognitive recovery after liver transplantation in cirrhotic patients. While the study is excellent, the manuscript could use some more clarity regarding the analysis pipeline, which is difficult to follow. Further, many citations are outdated and the neurocognitive mechanisms underlying the observed effects are described too vaguely, with the exception of the cerebellum. More recent citations and greater precision regarding the role of the brain regions and networks described is needed to better integrate the results in the literature. Furthermore, the grammar and overall language could be improved in many instances and I would hence recommend the manuscript to be proofread, especially the Discussion. In the following I detail some issues that me and the reviewers believe should be addressed.

Major

Introduction

- The authors should briefly comment on which specific aspects of neurocognitive functioning are most affected in cirrhotic patients, even if this is a data-driven study and even if neurocognitive deficits are present across multiple domains in cirrhosis or hepatic encephalopathy. Are the impairments most marked in memory, planning, language, motor, or other functions? Are these impairments mirrored by e.g. lower task-related activation, atrophy, or other neurological indicators? This would help to situate the present study in the literature, and more precisely delimit its contribution.

- The authors should briefly introduce Connectome-behavior predictive mapping (CPM) already in the Introduction. Better yet, a brief overview of the analysis pipeline would be helpful.

- Lines 77-78: The first and main research question is not formulated clearly, “1) are the disrupted or altered functional connections in cirrhotic patients with OHE or in those without?”. The authors should be more precise here, perhaps breaking down the question: do they expect altered connectivity to be altered in both groups relative to healthy controls? Do they expect differences between OHE and non-OHE patients?

Methods

- Lines 91-92: “in this study we evaluated functional connectivity and specifically its predictive power for behavior.” The authors should be more specific by what they mean with “behavior” here in order to better delineate the contribution of this manuscript.

- Neuropsychological tests: more details should be provided regarding the psychometric properties of the tests, e.g. split-half, internal consistency, and/or test-retest reliability if available from this dataset or from the literature. Indeed, the reliability of a measure is of paramount importance if it intends to measure inter-individual differences: https://pubmed.ncbi.nlm.nih.gov/28726177/

- CPM: the authors seem to describe “positive” and “negative” values in terms of their association with behavior (lines 211-215), but I can think of two scenarios that are not explicitly addressed, namely, negative connectivity values associated with better task performance and positive connectivity values associated with worse performance. The authors should more precisely explain how the method handles positive and negative connectivity values and the corresponding associations with better or worse neurocognitive performance.

- On a related note: why was positive network strength calculated as average edge strength and negative network strength as simply the sum of the r values? This decision must be clearly justified.

- The correspondence between methods and results is unclear and forces the reader to go back and forth between both sections. For instance, NBS is described before CPM but is reported in the opposite order in the Results section. A written and graphical overview of the analysis pipeline (similar to Figure 1 or Figure 5a) would be useful.

- Please provide more details on the cross-validation procedure.

- Line 226 refers to figure 4C but there is no section C in figure 4.

Results

- Lines 264-265: “decreased connectivity was mostly found in the connections among sub-cortical nuclei such as Amygdala, Putamen, Pallidum and orbital fontal cortex, as well as insula and cingulate cortex (Figure 2A).” The latter three regions are not subcortical, please rephrase.

- Table 1 should also include effect sizes (X2, t-, and F-values) and the caption could more succinctly depict the tests, e.g. . “c, d The p-value was obtained by the two-sample t test between pre-LT in non-OHE/OHE group and HCs (two-tailed)” could be written as “Two-tailed p-value for t-test patients ≠ controls”. Also, the acronym LT should be indicated.

- Line 364: please introduce the acronym PMN.

- The software and brain template used to present the connectivity results should be indicated.

Discussion

- Line 450, please rephrase with proper grammar, explaining more exactly what is meant: “Paracentral cortex is a well-known sensorimotor cortex.”

- More questionable writing and scholarship follows in lines 458-462: “Activities in the orbitofrontal cortex are associated with some higher order cognitive functions like learning (Schoenbaum and Roesch, 2005), rewarding (O’Doherty et al., 2001) and motivation (Arana et al., 2003). In the PMN, orbitofrontal cortex may play a role in the coordination of motor and higher order cognitive functions such as leaning and executive functions.” First of all, typos: “activities” cannot be plural, and “rewarding” is not a cognitive function. Second of all, please be more precise as to what is meant and cite more recent studies, as these are clearly outdated and the role of the orbitofrontal cortex is currently recognized to be more nuanced than what the authors portray.

- “bigger” -> better

Minor

- Although the manuscript is acceptably written, there are multiple typos that could be solved simply by applying language proofing available in most standard text processing programs.

- Line 46: “Successful LT can restore liver function completely, however,” -> “Successful LT can restore liver function completely. However,"

- Lines 61, 64, and others: “relavent” -> relevant

- Line 70: “FCs” -> FC

- Line 78: “connecitons” -> connections

- Line 481: “no presence” -> absence

- Line 495: “possibly” -> possible

## Author Response

July 19th, 2021

Dear editor of eNeuro,

This is the resubmission letter in response to the reviews of our manuscript (ID: eN-NWR-0114-21) entitled Neuromarkers from whole-brain functional connectivity reveal the

cognitive recovery scheme for overt hepatic encephalopathy after liver transplantation.

We thank the editor and reviewers for their careful consideration of our manuscript and

the helpful suggestions provided. We also thank the editor for his personal attention, and

the opportunity to re-submit our revised manuscript. We welcomed the opportunity to

further improve our manuscript based upon the feedback from reviewers. In light of all

comments, substantial revisions were conducted and included in this new version of the

manuscript. Please find specific revisions listed below, with the reviewers’ suggestions in

bold along with our responses to each. Sections of the manuscript that have been

updated to address these suggestions are highlighted in yellow in manuscript.

Major

Introduction

- The authors should briefly comment on which specific aspects of neurocognitive

functioning are most affected in cirrhotic patients, even if this is a data-driven study

and even if neurocognitive deficits are present across multiple domains in cirrhosis or

hepatic encephalopathy. Are the impairments most marked in memory, planning,

language, motor, or other functions? Are these impairments mirrored by e.g. lower

task-related activation, atrophy, or other neurological indicators? This would help to

situate the present study in the literature, and more precisely delimit its contribution.

We thank the reviewer for the suggestion to improve the manuscript. Accordingly, we

added the following text in the Introduction:

"Previous studies (Sotil et al., 2009; Bajaj et al., 2009) consistently demonstrate that

patients with cirrhosis both with and without OHE present low performance in Mini-Mental State Examination (MMSE), Number Connection Test (NCT) and digit symbol test

(DST). This suggests that the impairment led by cirrhosis is not limited to a specific

functional domain, but rather includes a wide range of cognitive functions including

short-term memory (recall), attention, language, comprehension, motor skill, as well as

executive functions. These impairments were reflected by brain atrophy and decreased 2

functional connectivity (Chen EJR 2012). “

Sotil, Eva U., et al. “Impact of preoperative overt hepatic encephalopathy on neurocognitive

function after liver transplantation.” Liver Transplantation 15.2 (2009): 184-192.

Bajaj, Jasmohan S., James B. Wade, and Arun J. Sanyal. “Spectrum of neurocognitive

impairment in cirrhosis: implications for the assessment of hepatic

encephalopathy.” Hepatology 50.6 (2009): 2014-2021.

Chen, Hua-Jun, et al. “Structural and functional cerebral impairments in cirrhotic patients with a

history of overt hepatic encephalopathy.” European Journal of Radiology 81.10 (2012): 2463-

2469.

- The authors should briefly introduce Connectome-behavior predictive mapping

(CPM) already in the Introduction. Better yet, a brief overview of the analysis pipeline

would be helpful.

Given the key role of CPM in our study, we agree that a brief overview of the method in

Introduction would be helpful for the audience to better understand. We have added a

short paragraph in Introduction:

"To avoid limiting our study to the analysis of brain regions, connections, and functional

networks hypothetically related to neurocognitive impairment in patients with cirrhosis,

we used “Connectome predictive mapping” (CPM) to identify unique patterns

characterizing cognitive performance. The CPM searches for all possible pairs of brain

regions and their associated connectivity values, consequently constructing a connectome

model that optimizes the association with behavioral scores (Finn et al., 2015; Rosenberg

et al., 2017; Shen et al., 2017). The predictive power and the robustness of the

connectome was further validated using cross-validation on novel/unseen subjects. As

such, CPM is a data-driven, prediction method that can identify what aspects of network

properties characterize cognitive performance in individuals.”

- Lines 77-78: The first and main research question is not formulated clearly, “1) are

the disrupted or altered functional connections in cirrhotic patients with OHE or in

those without?”. The authors should be more precise here, perhaps breaking down

the question: do they expect altered connectivity to be altered in both groups

relative to healthy controls? Do they expect differences between OHE and non-OHE

patients?

We agree with the reviewer that the question is not very clear. OHE remains the

most prevalent of visible neurological signs (Bajaj et al., Hepatology, Vol. 50, No. 6, 2009),

but previous studies also found brain structural and functional changes in patients with3

OHE as well as without. Given this evidence, we modify the question to: “compared to

healthy controls, are the disrupted or altered functional connections in cirrhotic patients

with OHE or in both cirrhotic groups regardless of OHE?”

Methods

- Lines 91-92: “in this study we evaluated functional connectivity and specifically its

predictive power for behavior.” The authors should be more specific by what they

mean with “behavior” here in order to better delineate the contribution of this

manuscript.

We apologize for the confusion. Behavior specifically indicates psychomotor cognitive

performance score; we have clarified this in manuscript.

- Neuropsychological tests: more details should be provided regarding the

psychometric properties of the tests, e.g. split-half, internal consistency, and/or test-retest reliability if available from this dataset or from the literature. Indeed, the

reliability of a measure is of paramount importance if it intends to measure inter-individual differences: https://pubmed.ncbi.nlm.nih.gov/28726177/

The neuropsychological tests involved in our study, namely the psychometric hepatic

encephalopathy scores (PHES), have been recommended for the assessment of

neuropsychological status in cirrhotic patients with and without overt HE by a working

party commissioned by the International Society for Hepatic Encephalopathy and

Nitrogen Metabolism (ISHEN). The PHES is composed of five tests, number connection

test-A (NCT-A), number connection test-B (NCT-B), serial dotting test (SDT), line tracing

test (LTT) and digit symbol test (DST). The PHES and the subtest scores correlate

significantly with the glucose utilization in brain regions involved in attention processes in

patients with minimal (mHE) or overt HE (Lockwood et al. 2002) and is internationally

recommended as the gold standard for the diagnosis of mHE.

The DST and NCT (as subtests in PHES) are simple tools for screening mHE among

inpatients with liver cirrhosis and have undergone repeated and rigorous psychometric

validation such as test-retest reliability and discriminant validity in a range of patient

samples (Matarazzo et al., 1984; Jaeger et al., 2018; Zeng et al., 2020). We thus selected

these tools for neuropsychological evaluation in our study.4

- CPM: the authors seem to describe “positive” and “negative” values in terms of

their association with behavior (lines 211-215), but I can think of two scenarios that

are not explicitly addressed, namely, negative connectivity values associated with

better task performance and positive connectivity values associated with worse

performance. The authors should more precisely explain how the method handles

positive and negative connectivity values and the corresponding associations with

better or worse neurocognitive performance.

We apologize for the ambiguous description. Positive and negative values in CPM were

defined as Pearson’s correlation coefficient r-value in terms of their correlation with

behaviors, regardless of better or worse performance. In our psychomotor cognitive tasks,

a higher DST score represented better performance, whereas higher NCT scores represent

worse performance. Hence, a positive r-value in CPM for DST score explains stronger

functional connectivity that is related to better cognitive performance. Whereas a

negative r-value for NCT score explains stronger functional connectivity related to better

performance. We have clarified this in the Methods.

- On a related note: why was positive network strength calculated as average edge

strength and negative network strength as simply the sum of the r values? This

decision must be clearly justified.

We apologize for the confusion; both positive and negative network strengths are

calculated by averaging the r values over the edges/connections selected from CPM. We

have corrected this in our new manuscript.

- The correspondence between methods and results is unclear and forces the reader

to go back and forth between both sections. For instance, NBS is described before

CPM but is reported in the opposite order in the Results section. A written and

graphical overview of the analysis pipeline (similar to Figure 1 or Figure 5a) would be

useful.

We thank the reviewer for the suggestion. Accordingly, we reordered the result section to

make a stronger coherence between Methods and Results. We also added a written

overview of the NBS analysis pipeline in Methods section, which is also described below.

"NBS was first applied to compare the FC differences between controls and pre-LT

patients to show the disrupted connectivity induced by cirrhosis. NBS was then applied to

compare controls and post-LT to show whether there was still impaired FC after the LT. 5

Finally, NBS was conducted to compare pre-LT and post-LT to investigate the FC recovery

and hyperconnectivity.”

- Please provide more details on the cross-validation procedure.

CPM was performed using a 5-fold cross validation. Specifically, all control participants

were randomly split into 5 subgroups. Among the 5 groups, one group was selected as

the test dataset and the remaining 4 groups were used to train a CPM model to search for

predictive functional connectivity for each of the cognitive task scores (DST, NCT). For the

training, a linear regression model was used to fit the average value of obtained

functional connectivity with each cognitive performance score. This trained model was

then applied to predict performance scores in the test dataset. This process repeated 5

times until all 5 groups were tested. To validate the predictive accuracy and the

robustness of the functional connectivity identified by CPM models, we correlated

predicted performance scores with the observed performance scores for all participants. If

the correlation was reported to be significant, then the consensus of functional

connectivity maps obtained from all 5 CPM models was generated as the Psychomotor

network (PMN).

- Line 226 refers to figure 4C but there is no section C in figure 4.

Sorry for the confusion. We have removed the citation of figure 4C from our manuscript

as it does not exist.

Results

- Lines 264-265: “decreased connectivity was mostly found in the connections among

sub-cortical nuclei such as Amygdala, Putamen, Pallidum and orbital fontal cortex, as

well as insula and cingulate cortex (Figure 2A).” The latter three regions are not

subcortical, please rephrase.

Thanks for the comment to improve the text. Accordingly, we have modified it as:

"decreased connectivity was mostly found in the connections among subcortical nuclei

such as Amygdala, Putamen, Pallidum as well as cortical regions including orbital fontal,

insular and cingulate cortices"6

- Table 1 should also include effect sizes (X2, t-, and F-values) and the caption could

more succinctly depict the tests, e.g. . “c, d The p-value was obtained by the two sample t test between pre-LT in non-OHE/OHE group and HCs (two-tailed)” could be

written as “Two-tailed p-value for t-test patients ≠ controls”. Also, the acronym LT

should be indicated.

We have updated Table 1 by including the effect sizes.

- Line 364: please introduce the acronym PMN.

PMN represents Psychomotor network, which has been clarified in text.

- The software and brain template used to present the connectivity results should be

indicated.

Functional connectivity was visualized using BrainNetViewer toolbox in MATLAB, and an

MNI-ICBM152 brain template (http://www.bic.mni.mcgill.ca/ServicesAtlases/

ICBM152NLin2009) was used to show the brain coordinates. We have added this

information into our new manuscript.

Xia, Mingrui, Jinhui Wang, and Yong He. “BrainNet Viewer: a network visualization tool for human

brain connectomics.” PloS one 8.7 (2013): e68910.

Discussion

- Line 450, please rephrase with proper grammar, explaining more exactly what is

meant: “Paracentral cortex is a well-known sensorimotor cortex.”

We corrected the text as:

"Paracentral cortex is located on the medial surface of the cerebral hemisphere and is the

medial continuation of the precentral and postcentral gyri. It plays an important role in

motor and sensory functions related to the contralateral lower limb.”

- More questionable writing and scholarship follows in lines 458-462: “Activities in

the orbitofrontal cortex are associated with some higher order cognitive functions

like learning (Schoenbaum and Roesch, 2005), rewarding (O’Doherty et al., 2001) and

motivation (Arana et al., 2003). In the PMN, orbitofrontal cortex may play a role in 7

the coordination of motor and higher order cognitive functions such as leaning and

executive functions.”

First of all, typos: “activities” cannot be plural, and “rewarding” is not a cognitive

function. Second of all, please be more precise as to what is meant and cite more

recent studies, as these are clearly outdated and the role of the orbitofrontal cortex is

currently recognized to be more nuanced than what the authors portray.

We thank the reviewer for indicating the grammar issues, which we have corrected in our

new manuscript. Also, we’ve changed the paragraph above to below:

"The activity in the orbitofrontal cortex is associated with some higher order cognitive

functions like emotion and emotion regulation (Rolls et al., 2020), executive function

(Nejati et al., 2018), reward learning (Izquierdo, 2017) and decision making (Du et al.,

2017). Particularly, a recent study also indicated that the OFC encodes decision variables

and instructs sensory areas to guide adaptive behavior (Banerjee et al., 2020). In the PMN,

it is possible that the OFC plays an important role in guiding adaptive behavior to achieve

a cognitive task goal in an indirect and sophisticated way.”

Du, Jingnan, et al. “Functional connectivity of the orbitofrontal cortex, anterior cingulate cortex,

and inferior frontal gyrus in humans.” Cortex 123 (2020): 185-199.

Izquierdo, Alicia. “Functional heterogeneity within rat orbitofrontal cortex in reward learning and

decision making.” Journal of Neuroscience 37.44 (2017): 10529-10540.

Nejati, Vahid, Mohammad Ali Salehinejad, and Michael A. Nitsche. “Interaction of the left

dorsolateral prefrontal cortex (l-DLPFC) and right orbitofrontal cortex (OFC) in hot and cold

executive functions: Evidence from transcranial direct current stimulation

(tDCS).” Neuroscience 369 (2018): 109-123.

Rolls, Edmund T., et al. “Functional connectivity of the right inferior frontal gyrus and orbitofrontal

cortex in depression.” Social cognitive and affective neuroscience 15.1 (2020): 75-86.

Banerjee, Abhishek, et al. “Value-guided remapping of sensory cortex by lateral orbitofrontal

cortex.” Nature 585.7824 (2020): 245-250.

- “bigger” -> better

This has been corrected in new manuscript.

Minor (Mengting)

- Although the manuscript is acceptably written, there are multiple typos that could 8

be solved simply by applying language proofing available in most standard text

processing programs.

One of the authors (J.S.) is an English native speaker, has run language proofing, checked

and corrected the grammar and typo issues along with all the suggestions from the

reviewer.

- Line 46: “Successful LT can restore liver function completely, however,” ->

"Successful LT can restore liver function completely. However,"

Corrected.

- Lines 61, 64, and others: “relavent” -> relevant

Corrected.

- Line 70: “FCs” -> FC

Corrected.

- Line 78: “connecitons” -> connections

Corrected.

- Line 481: “no presence” -> absence

Corrected.

- Line 495: “possibly” -> possible

Corrected
